# Effects of augmented reality cueing strategies on freezing of gait: The ELIMINATE FoG trial

**DOI:** 10.1016/j.prdoa.2025.100332

**Published:** 2025-04-29

**Authors:** Brendan Baugher, Ryan Kaya, Claire Sonneborn, Kenneth B. Baker, Hubert H. Fernandez, Nathaniel Szewczyk, Jay L. Alberts, James Liao

**Affiliations:** a Heritage College of Osteopathic Medicine, Ohio University, 4180 Warrensville Center Rd, Warrensville, OH 44122, United States; b Center for Neurological Restoration, Neurological Institute, Cleveland Clinic, 9500 Euclid Ave, Cleveland, OH 44195, United States; c Russ College of Engineering and Technology, Ohio University, 28 W Green Dr, Athens, OH 45701, United States; d Quantitative Health Sciences, Lerner Research Institute, Cleveland Clinic, 9500 Carnegie Ave, Cleveland, OH 44106, United States; e Neuroscience, Lerner Research Institute, Cleveland Clinic, 9500 Carnegie Ave, Cleveland, OH 44106, United States; f Ohio Musculoskeletal and Neurological Institute, Ohio University, Athens, OH 45701, United States; g Biomedical Engineering, Lerner Research Institute, Cleveland Clinic, 9500 Carnegie Ave, Cleveland, OH 44106, United States

**Keywords:** Parkinson’s disease, Gait freezing, FoG, Augmented reality, Cue

## Abstract

**Background:**

Freezing of gait (FoG) is a treatment-resistant symptom of Parkinson disease (PD). Augmented reality (AR) cues have been investigated as a therapy for FoG, with inconclusive results from a limited array of AR constructs.

**Objectives:**

Compare four modalities of a novel AR cue to physical and no-cue controls.

**Methods:**

Presence of FoG in PD was required; exclusion criteria included dementia, severe vision loss, and significant gait-disrupting comorbidities. Participants completed six walking tasks, featuring different cueing conditions in a crossover fashion, in a holographic hallway displayed by an AR headset. A conventional physical cue was presented first, followed by other conditions in randomized order (*hand-controlled* AR cue, *observer-controlled* AR cue, *eye-controlled* AR cue, *constant* AR cue, no-cue control). Primary outcomes were FoG duration and incidence, manually annotated. Secondary outcomes included survey questions and gait parameters derived from IMUs.

**Results:**

Thirty-six participants completed testing. The *observer-controlled* AR cue produced lower FoG duration than the no-cue, physical, and *hand-controlled* AR cue conditions (N = 36, p ≤ 0.006, Wilcoxon effect size (WES) ≥ 0.46). The *constant* cue reduced FoG incidence compared to all other conditions (N = 36, p ≤ 0.016, WES ≥ 0.40). Participants’ preferred AR cues decreased FoG duration (N = 28, p ≤ 0.004, WES ≥ 0.48) and incidence (N = 28, p ≤ 0.022, WES ≥ 0.38) compared to controls. Differences in kinematic outcomes were negligible. Survey results indicated receptiveness toward AR cueing, with diversity in preferred cue activation modalities. No significant adverse events occurred.

**Conclusions:**

AR cueing decreased FoG incidence and duration compared to controls. Efficacy of discrete cueing modalities likely depends on user intrinsic factors, such as preference.

## Highlights

• A multidirectional AR cue was created to address FoG on straight paths *and* turns.
• Four different modalities of the cue were tested: one constant and three as-needed.
• On-demand, observer-driven AR cues reduced FoG duration.
• Constantly-on AR cues reduced FoG incidence.
• Preference matters: favored modalities outperformed discrete conditions.

## 1 Introduction

Freezing of gait (FoG) is a Parkinson’s disease (PD) symptom that reduces quality of life and causes falls [1]. FoG impacts over half of people with advanced PD and is characterized by reduction or cessation of gait despite intent to walk. FoG varies in phenotype (broadly, trembling vs akinetic) and triggers (e.g. sensory and/or cognitive distractions) [2,3]. It often occurs at doorways, turns, or when initiating gait, but can also be seemingly random [4]. FoG produces anxiety about ambulation due to fear of falling and social stigma. This mental burden may induce FoG, perpetuating a harmful cycle [2,3].

FoG is often resistant to dopaminergic medications and deep brain stimulation (DBS), and in some cases may be exacerbated by these therapies [1,5]. External cues are an alternate method of combatting FoG. Cues are thought to promote goal-directed stride maintenance and/or rescue guidance via activation of alternate neural gait-control pathways [6,7]. Examples of existing cues include stride-synchronized sounds, physical lines, and laser projections [[8], [9], [10]].

Augmented reality (AR) offers unique potential for delivering versatile, dynamic cues not limited by physical constraints. Despite this, AR cues have largely been modeled after a physical cue: lines perpendicular to walking paths [9]. Exploratory trials have mostly investigated the effects of AR cues on gait parameters like speed, with mixed results in individuals with FoG [[11], [12], [13], [14], [15], [16], [17]]. Few AR trials have measured FoG directly; those that did observed no beneficial effects [11,13,14,18]. This may reflect inferiority of AR cues to physical cues, or technical limitations of AR hardware (e.g. small field-of-view) that counteract any gait benefits. It is also possible that prior studies were underpowered or used paradigms that did not reliably trigger FoG [3,19].

In this study, a novel AR cue was tested in a course designed to trigger FoG. The cue was circumferential, intended for straight paths *and* turns. The cue was projected by a commercially available headset, the Magic Leap 2 (ML2), with a larger field-of-view than previously studied headsets [13,18]. Specific aims were to: 1) compare cue-responsiveness between AR and physical cueing; 2) test constant vs on-demand AR cueing; 3) compare three methods of intermittent, on-demand cue activation; 4) assess the effects of all cueing methods against a no-cue control. We hypothesized an externally-activated intermittent cue would be more effective than user-controlled intermittent cues due to less cognitive load with the former.

## 2 Methods

### 2.1 Participants

Thirty-six patients with PD-FoG were recruited (see Supplemental Document 1 for sample estimate). Presence of FoG was determined by scores > 0 on the most recently documented Movement Disorder Society-Unified Parkinson’s Disease Rating Scale (MDS-UPDRS) items 2.13 (“Over the past week, on your usual day when walking, do you suddenly stop or freeze as if your feet are stuck to the floor?”) or 3.1 (clinical observation of FoG) [20]. FoG was confirmed at screening with the New Freezing of Gait Questionnaire (NFOG-Q) [21]. Participants were also evaluated at screening with the motor component (section III) of the MDS-UPDRS and Montreal Cognitive Assessment (MoCA), in their normal medication state (ON, if prescribed PD medications) [21,22].

Exclusion criteria included significant gait comorbidities (e.g. severe orthopedic conditions), inability to walk without an assistive device, severe bilateral visual impairment, age <21, or dementia diagnosis. DBS was allowed if persistent FoG was reported despite stimulation. Those with stimulators were instructed to use the device as usual during testing.

Initially, PD medications were held on test day to increase likelihood of FoG. This limited participation for individuals with OFF symptoms severe enough to make travel unsafe. An amendment was made to allow PD medications if participants reported inability to participate OFF medications but experienced FoG ON medication. The MDS-UPDRS Part 3 was repeated on test day ON or OFF medication, dependent on this criteria.

Trial protocol and recruitment were approved by the Cleveland Clinic IRB and registered on ClinicalTrials.gov (NCT05608915). All participants provided written informed consent.

### 2.2 Cueing interventions

**Fig. 1:**
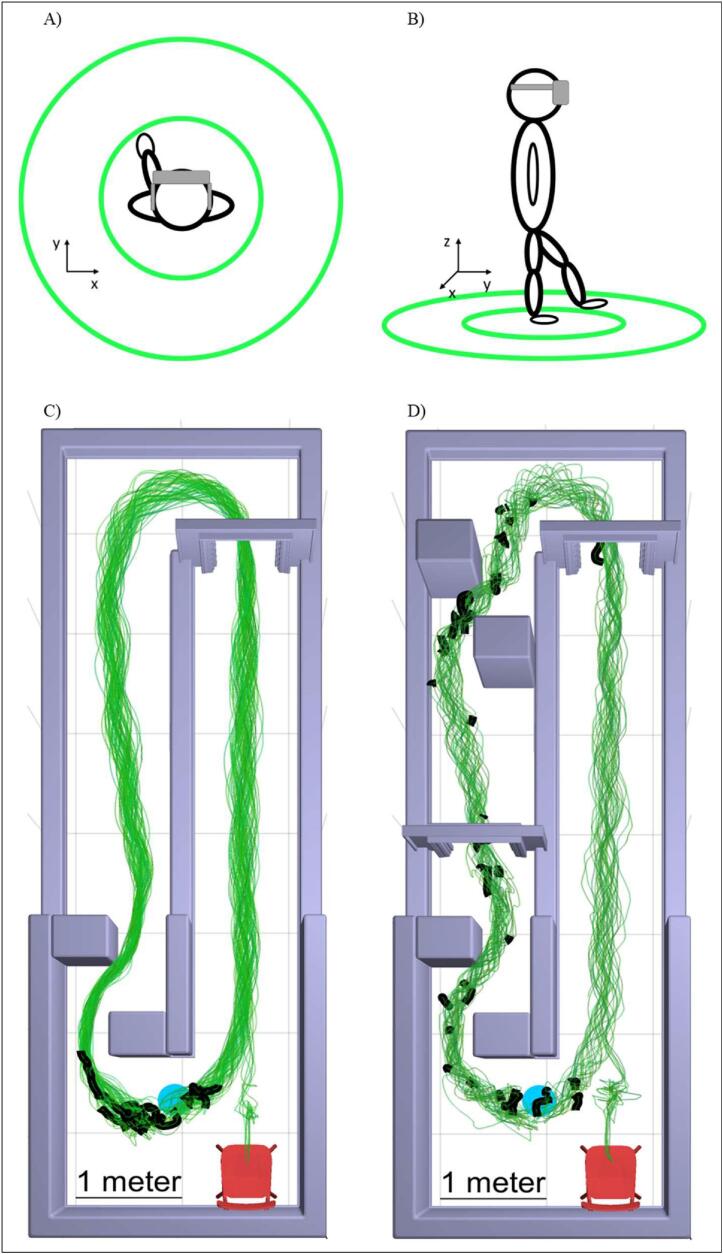
Aerial (A) and lateral (B) views of the novel AR cue, and 3D models of course Level 1, 2 (C) and 3 (D) from aerial view. Green traces represent the paths of two participants throughout all six conditions. Thick black traces indicate FoG episodes. Blue circles represent the pivot circle. Columns and tall walls were 2.5 m high. Short walls were 0.5 m high. The interior width of the doorways was 0.55 m, and the distance between columns was 0.5 m. The hallway itself was 7.5 m long and 2.2 m wide.

Six cueing conditions were tested: four AR conditions, a physical cue, and a no-cue control. The AR cue consisted of two concentric rings (Fig. 1A,B) with radii 40 % and 80 % of body height, based on previously established physical cues [9,23]. Each ring was 2 in. wide and neon green (high visibility), with no appreciable height.

In the ***constant*** AR condition, the cue was present throughout the walking task. It moved with the user like a “halo” on the ground, centered on the projection of head position onto the floor.

The other three AR conditions were intermittent or “as-needed”: *eye-controlled*, *hand-controlled*, and *observer-controlled*. The former two were user-activated, while the latter was externally activated. These intermittent cues did not follow the participant, remaining static where activated. Participants could step beyond the rings, which would then disappear until re-triggered.

In the ***eye-controlled*** condition, the cue was triggered when the ML2 detected the user’s gaze vector on the ground within a circle of radius 0.8x body height centered on the user (the same radius as the cue’s outer ring). The outer ring remained invisible until the gaze vector aligned with this predefined zone. This was based on evidence that people with PD tend to look down when walking [24].

The *hand-controlled* and *observer-controlled* AR conditions were activated by a Bluetooth-connected clicker. In the ***hand-controlled*** AR condition, participants carried the clicker and could activate the cue ad libitum.

In the ***observer-controlled*** AR condition, an investigator activated the cue when they observed FoG. This was a stand-in for a reliable FoG detection/prediction algorithm, development of which is an ongoing effort of the research community [25].

The **physical** cue consisted of parallel white lines of non-reflective tape, perpendicular to straight sections of the course. These were spaced at 40 % of body height similar to the AR cue [9,23]. Finally, a **no-cue** control condition was included.

AR cues were constructed in a 3D engine (Unity Software, Inc., San Francisco, CA, USA) and configured with a custom iPad (Apple, Cupertino, CA, USA) app controlling cue radius, cue condition, and dual-tasks (see below).

### 2.3 Experimental task

Testing was conducted in a holographic hallway course (Fig. 1C and D) displayed by the ML2, worn in all conditions. The hallway included straight segments divided by a quarter-wall with an arc-turn on one end and a pivot-turn on the other. FoG-instigating features included one or more doorways (depending on difficulty level) and pillar-like obstacles. A holographic environment was chosen so investigators would not be inhibited in providing aid in cases of instability.

Participants began in a chair, then proceeded through the course in a counter-clockwise fashion, turning 180° at a holographic pivot circle, and proceeding back through the course clockwise, turning at the circle and moving counter-clockwise, etc. This was repeated, with an upper limit of five minutes of walking, for each condition. More time was allowed if participants were nearing the starting point at five minutes; testing was ended early if participants became fatigued/unstable. The minimum time that constituted a completed condition was one minute.

Three levels of difficulty were available. Level 1 featured a single doorway and two column-like obstacles (Fig. 1C). Level 2 added an auditory cognitive task through the ML2, either simple math or reverse spelling (i.e. “spell truck backward”). Participants were instructed to answer quickly to the best of their ability. Level 3 added another doorway and two more obstacles along with cognitive tasks (Fig. 1D).

### 2.4 Trial design

This single-center, crossover, open-label, exploratory trial assessed novel interventional methods of combatting FoG. One screening and one testing visit were required. Participants were unblinded due to the visual/intentional nature of the cues. Investigators were unblinded due to testing artifacts (presence of the physical cue or clicker in three of six conditions).

Before testing, participants completed three activities to acclimate to the equipment (six inertial measurement units (IMUs) and the ML2), tasks, and cues. Participants were instructed to walk at a comfortable speed. Acclimation 0 included two 5 m walks without equipment to measure baseline speed. The ML2 (turned off) and IMUs were added for Acclimation 1, where participants performed 5 m walks until their speed was within 5 % of Acclimation 0.

The ML2 and holographic hallway were turned on for Acclimation 2, difficulty was then titrated. If <5 FoG episodes were observed in one “round” (navigation through the course to the turning indicator, then back to the starting point) at Level 1, difficulty was increased to Level 2, and if needed, Level 3. The highest level needed was used for formal testing. Rounds were repeated until completion time was within 5 % of the previous round or decreased then increased (suggesting fatigue). In the final step of Acclimation 2, cueing conditions were introduced. Participants were instructed to use the cues however they saw fit; examiners stated that the cues were intended to help combat FoG and that similar cues have been used as a guiding path or marker to step on/over.

Testing included six conditions, each separated by approximately five minutes of rest. The physical cue was used to align the course, so was present starting in Acclimation 2. Placing the physical cue and orienting the course necessitated an investigator wear the headset, potentially adding a uniquely long interruption between the physical cue and other conditions. To address this, the physical cue was set up before participant arrival and was the first condition presented. It was removed for the remaining conditions, which were presented in an unrestricted, randomized sequence generated for each participant using a custom MATLAB script.

### 2.5 Data collection and analysis

Kinematic data was collected from the ML2 and six APDM Opal IMUs via MobilityLab (APDM Wearable Technologies, Portland, OR, USA) software (one per wrist, one per ankle, one around the waist, and one attached to the ML2). IMUs were synchronized to the ML2 in MATLAB R2022b using intentional motion artifacts in both systems. ML2 data was upsampled to 128 Hz to match the IMUs. Gait videos were recorded via tripod-mounted iPad for later offline annotation. Participants were surveyed about their overall experience and cue preferences (see *Survey*).

### 2.6 Data availability statement

De-identified data from this study may be released upon application to the Cleveland Clinic Institutional Review Board by contacting 216-444-2924 or IRBHelp@ccf.org.

### 2.7 Primary outcomes

Primary outcomes included percent time frozen and freeze rate. Percent time frozen represents time frozen/total task duration. Time frozen and gait task duration were derived from video annotation, the current gold standard for assessment of FoG [3]. Annotation from a single, principal grader was used. Manual annotation was completed via custom MATLAB R2022b scripts. To assess grading reliability, annotations were compared to a secondary grader in a reliability subset (N = 3). Agreement on time frozen for this subset was 99.63 % (3570.6 s of FoG documented by principal grader).

Freeze rate represents the number of FoG episodes per minute. This was also derived from video annotation. Agreement between graders in the reliability subset was 83.44 % (151 instances documented by principal grader). Decreased agreement compared to time spent frozen was due to instances where graders disagreed on whether episodes constituted one long freeze or several freezes broken by short FoG-free periods.

### 2.8 Secondary outcomes

Secondary outcomes included gait metrics and survey responses. The survey was added after the first two participants completed testing, though they did provide documented commentary that answered some survey questions (e.g. most/least effective conditions). Gait metrics included stride time coefficient of variation, stride length, and speed.

To assess step timing, an established method to detect peak angular velocity during the swing phase of each foot was used [26]. Each ankle’s IMU gyroscope detected angular velocity during gait, continuously. To find peak angular velocities, we first applied a 12th-order Butterworth low-pass filter with 8 Hz cutoff-frequency to filter the magnitude of angular velocity using MATLAB’s filtfilt function. Peaks were identified using MATLAB’s islocalmax function, with minimum prominence 1, minimum separation 0.5 s, and prominence window 1 s. Stride times are the time from one peak velocity to the next, of the same foot. Times from both feet were pooled. Stride time coefficient of variation was calculated as standard deviation of stride duration/mean stride duration [27].

Stride length was derived from the ML2 headset, which detects its position in global coordinates. ML2 headset position at velocity peaks (see above) were used to define each step’s position. Stride lengths are defined as the distances between successive step positions. Mean stride length is from pooled values of both feet. Mean speed is mean stride length/mean stride duration.

### 2.9 Statistics and reproducibility

Within-subjects comparisons were performed for each variable. Nonparametric primary outcomes were analyzed using Wilcoxon signed-rank tests, while paired t-tests were applied to secondary outcomes. Significance (alpha) was set at 0.05. Analyses were completed with MATLAB R2022b’s signed-rank and *t*-test functions. Effect sizes were determined using Cohen’s d for t-tests and Wilcoxon effect size for signed-rank tests. Given the exploratory study design, no corrections for multiple comparisons were applied. However, clinical significance was prioritized as events with significant p-values but small effect sizes (Cohen’s absolute value <0.30 or Wilcoxon effect size <0.10) were treated as insignificant [28].

A post-hoc subanalysis explored the relationship between cue preference and efficacy after noting significant variability in participant preferences for discrete cueing modalities. Participants selecting an AR cue as the most effective condition in the post-trial survey had their chosen condition added to a “preferred AR cue” group. This group was compared to physical and no-cue conditions using Wilcoxon signed-rank tests for primary and paired t-tests for secondary outcomes.

In another post-hoc analysis, the frequency of cue activation in response to FoG episodes was determined for the intermittent cues. Cues were considered associated with FoG episodes if they were already active at FoG onset, or if cues were activated within 5 s after FoG onset.

## 3 Results

### 3.1 Demographics

Thirty-eight participants were enrolled. One failed screening and another withdrew voluntarily, resulting in 36 participants completing testing. Recruitment and testing occurred from November 2022 to July 2023; there was no follow-up after testing. All participants on PD medication (N = 34) had FoG, per self-report, that was worse in the OFF medication state. Demographic profiles of participants who completed testing are summarized in Table 1.

**Table 1:** Demographic Information [N = 36] [PD = Parkinson’s disease, FoG = freezing of gait, DBS = deep brain stimulation].

Variable	Mean

Age	68.0 (SD 9.1)
New Freezing of Gait Questionnaire score	19.3 (SD 5.5)
Montreal Cognitive Assessment score	25.3 (SD 3.8)
Movement Disorder Society-Unified Parkinson’s Disease Rating Scale motor component (section III) at test visit	41.8 (SD 16.3)
Years since PD diagnosis	7.7 (range 0.3,20.3)

Variable	Frequency

Self-identified race:	
White	88.9 %
Declined	5.6 %
Asian	2.8 %
Black	2.8 %
Self-identified ethnicity:	
Non-Hispanic	94.4 %
Declined	5.6 %
Sex:	
Male	66.7 %
Female	33.3 %
Medication status for walk tests:	
ON (N=19)	52.8 %
OFF (N=15)	41.2 %
Not on PD meds at baseline (N=2)	5.6 %
Deep brain stimulator:	
Absent	75 %
Bilateral subthalamic nucleus, active	22.2 %
Bilateral globus pallidus internus, active	2.8 %
Titrated task difficulty:	
Level 1 (1 door, 2 obstacles)	58.3 %
Level 2 (1 door, 2 obstacles, dual-tasking)	25.0 %
Level 3 (2 doors, 4 obstacles, dual-tasking)	16.7 %

### 3.2 Walk test results

In total, 1,285 episodes of FoG were observed (median = 32.5(IQR 17.5,49)). Only 5.6 % of episodes began in the straight, unobstructed portion of the course, despite that portion being ∼31 % of the course. Participants on average walked 101.5 m(SD 54.0 m) over 199.2 s(SD 69.5 s) for each condition (excluded is one participant for whom kinematic data was lost). All participants completed at least one minute of testing per condition. Combining all participants, 98.08 % of freezes had associated cue activations in the *observer-controlled* condition, compared to 75.34 % in the *eye-controlled* condition and 63.86 % in the *hand-controlled* conditions. The other conditions did not require activation. Twenty-eight participants met the “preferred AR cue” criterion by selecting an AR cue as the most effective condition.

### 3.3 Primary outcomes (Fig. 2)

Percent Time Frozen

**Fig. 2:**
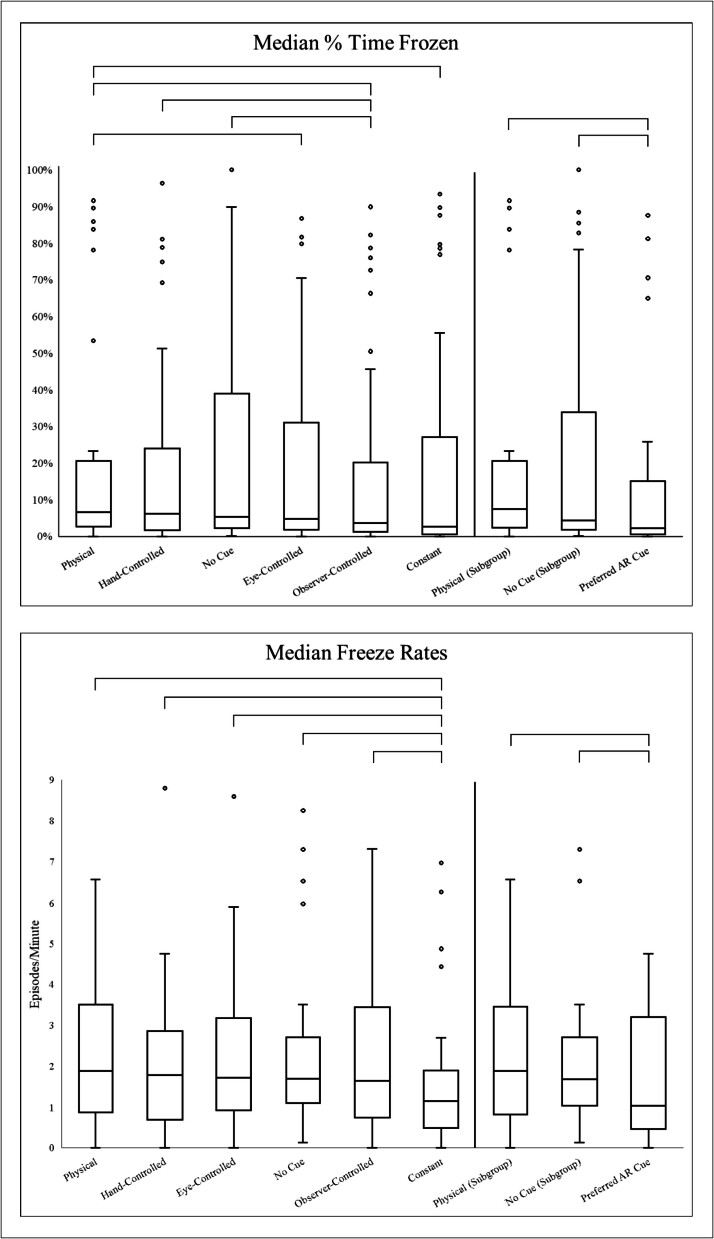
Percent time frozen (top) and freeze rates (bottom), per condition, ordered by descending medians. Significant differences in the conditions are denoted with brackets, with significance based on Wilcoxon signed-rank tests.

The *observer-controlled* condition resulted in less time frozen than the:

• No-cue condition (N = 36, p = 0.004, |Z| = 2.91, moderate Wilcoxon effect size (WES) = 0.48, median of differences (MoD) = −1.20 %(IQR −4.94 %,2.55 %))
• *Hand-controlled* cue (N = 36, p = 0.006, |Z| = 2.78, moderate WES = 0.46, MoD = −1.69 %(−5.39 %,2.01 %))
• Physical cue (N = 36, p = 0.003, |Z| = 2.96, moderate WES = 0.48, MoD = −3.14 %(−10.05 %,3.77 %))

In addition to being less effective than the *observer-controlled* cue, the physical cue resulted in more time frozen than the:

• *Constant* cue (N = 36, p = 0.027, |Z| = 2.21, moderate WES = 0.37, MoD = 2.03 %(−2.53,6.60 %))
• *Eye-controlled* cue (N = 36, p = 0.011, |Z| = 2.54, moderate WES = 0.42, MoD = 2.63 %(−2.67 %,7.94 %))

The preferred AR cue exhibited less time frozen than the:

• No-cue condition (N = 28, p = 0.004, |Z| = 2.87, moderate WES = 0.48, MoD = −1.53 %(−7.25 %,4.18 %))
• Physical cue (N = 28, p = 0.002, |Z| = 3.17, large WES = 0.53, MoD = −2.08 %(−8.59 %,4.43 %))

Freeze Rate

The *constant* cue had significantly lower rates of freezing than all other discrete conditions:

• No-cue condition (N = 36, p = 0.001, |Z| = 3.27, large WES = 0.54, MoD = −0.56(−1.64,0.52))
• *Eye-controlled* cue (N = 36, p = 0.002, |Z| = 3.11, large WES = 0.52, MoD = −0.44(−1.72,0.83))
• Physical cue (N = 36, p = 0.003, |Z| = 3.00, large WES = 0.50, MoD = −0.75(−1.93,0.43))
• *Hand-controlled* cue (N = 36, p = 0.012, |Z| = 2.51, moderate WES = 0.42, MoD = −0.45(−1.64,0.74)),
• *Observer-controlled* cue (N = 36, p = 0.016, |Z| = 2.41, moderate WES = 0.40, MoD = −0.13(−1.41,1.15))

Preferred AR cues exhibited lower freeze rates than the:

▪ No-cue condition (N = 28, p = 0.022, |Z| = 2.30, moderate WES = 0.38, MoD = −0.43(−1.41,0.55))
▪ Physical cue (N = 28, p = 0.009, |Z| = 2.62, moderate WES = 0.44, MoD = −0.41(−1.98,1.15))

Median values and all analyses for primary outcomes are included in Supplemental Tables 1 and 2, respectively.

### 3.4 Secondary outcomes

No significant differences in stride time coefficient of variation were found between any conditions.

Stride length was longer in the preferred cue (mean = 0.61 m, 95 % CI 0.54,0.68) than physical cue condition (mean = 0.56 m, 95 % CI 0.49,0.63) (t(26) = 3.30, p = 0.003). The effect size (d = 0.26) indicates this difference was not meaningful.

Speed was greater in the *observer-controlled* (mean = 0.51 m/s (95 % CI 0.45,0.57) than physical cue condition (mean = 0.48 m/s, 95 % CI 0.43,0.54) (t(34) = 2.70, p = 0.011). Speed was also greater in the preferred (mean = 0.54 m/s, 95 % CI 0.47,0.61) than physical cue (mean = 0.49 m/s, 95 % CI 0.43,0.56) (t(26) = 3.28, p = 0.003). Effect sizes (d = 0.15, d = 0.25) indicate these differences were not meaningful.

All means and comparisons for secondary outcomes are included in Supplemental Tables 3 and 4.

*Survey* (Table 2)

**Table 2:** Survey Results [FoG = freezing of gait; *these cues were used for the “preferred cue” sub-analysis].

Do you think the cue helps you to walk better? (N = 36)	
Yes	75 %
No	22.20 %
Unsure	2.80 %

Do you think the cue helps prevent freezing? (N = 36)	

Yes	75 %
No	22.20 %
Unsure	2.80 %

Did the cue help with freezing at doorways? (N = 34)	

Yes	70.60 %
No	14.70 %
Denied experiencing FoG in this location	14.70 %

Did the cue help with freezing when turning? (N = 34)	

Yes	88.20 %
No	8.80 %
Denied experiencing FoG in this location	2.90 %

Did the cue help with freezing at obstacles? (N = 34)	

Yes	64.70 %
No	20.50 %
Denied experiencing FoG in this location	8.80 %
Unsure	5.90 %

Did the cue help with freezing on straight paths? (N = 34)	

Denied experiencing FoG in this location	58.80 %
Yes	20.60 %
No	20.60 %

Which condition was the most effective?* (N = 36)	

Constant	25 %
Hand-controlled	25 %
Eye-controlled	13.90 %
Observer-controlled	13.90 %
Physical	11.10 %
Unsure	5.60 %
No cue	5.60 %

Which condition was the most difficult? (N = 36)	

No cue	30.60 %
Eye-controlled	22.20 %
Observer-controlled	16.70 %
Hand-controlled	13.90 %
Physical	5.60 %
Constant	5.60 %
Unsure	5.60 %

Did you prefer to have the cue present at the moment you freeze, or before? (N = 36)	

Before	66.70 %
Onset	27.80 %
Unsure	5.60 %

Was it easier to activate the cue when you wanted it with the clicker or with your eyes? (N = 36)	

Clicker	63.90 %
Eyes	36.10 %

Did you prefer having control over the cue or the examiner having control? (N = 36)	

Self-control	72.20 %
Observer-control	25 %
Indifferent	2.80 %

If this system was lighter, had a bigger field of view, a long battery life, and looked similar to regular glasses, would you use the cues in the real world on a daily basis? (N = 36)	

Yes	86.10 %
No	8.30 %
Unsure	5.60 %

### 3.5 Adverse events

No adverse effects were reported. Several patients experienced severe FoG episodes and required stabilization by investigators to prevent falls. One patient dropped to their knees to prevent a serious fall, suffering a minor abrasion. After resting briefly, they felt comfortable continuing.

## 4 Discussion

Visuospatial AR cues differentially reduced FoG in this cohort. The *observer-controlled* cue demonstrated a moderate reduction in percent time frozen compared to *hand-controlled*, physical, and no cues. The *constant* cue demonstrated a large reduction in freeze rate relative to all other discrete modalities (except a moderate effect compared to *observer-controlled*). Among participants who preferred AR cues, their preferred cue significantly outperformed no-cue and physical cue conditions in percent time frozen and freeze rate. The success of *observer-controlled* and *constant* cues, which required no user input, supports the hypothesis that lower cognitive demand improves efficacy. The *observer-controlled* cue was activated for nearly 100 % of FoG episodes, compared to ∼75 % for *eye-controlled* and ∼64 % for *hand-controlled* cues. This suggests participants either chose not to or could not activate the user-controlled cues for a substantial number of freezing events.

This study did not clarify whether intermittent or constant cues were superior. Intermittent cues decreased percent time frozen while the *constant* cue decreased freeze rate. It is worth considering why an intervention would reduce percent time frozen and not freeze rate, and vice versa. The *observer-controlled* cue, which reduced percent time frozen but not incidence of FoG, activated on FoG occurrence. It was suited to escaping rather than preventing FoG episodes, likely resulting in minimal effects on freeze rate. Conversely, the *constant* cue may have provided guidance that helped reduce FoG incidence, but may be insufficient to activate goal-directed pathways that allow *escape* from FoG. Thus, constant cueing would have weaker effects on freeze duration.

This explanation reflects contemporary thoughts on non-AR cueing. Constant cues are considered suboptimal to intervene in persistent FoG due to habituation [29], and reactionary on-demand cues have demonstrated more efficacy in decreasing FoG duration, not incidence [30]. Few, and heterogenous, AR cueing studies have measured these FoG-specific outcomes [11]. FoG phenotype also likely influenced results. Some FoG phenotypes include only brief episodes, resulting in a low percentage time frozen even with high incidence. The varying efficacy of AR cueing based on FoG phenotype warrants further investigation, as previous studies have identified phenotype-specific responses to non-AR cues [30].

The *observer-controlled* and preferred cues, which decreased FoG duration, may have paradoxically increased incidence by segmenting longer freezes into shorter episodes. For instance, one participant froze for the full duration of a task without cues (FoG episodes = 1) but was FoG-free between shorter freezing episodes with cues (FoG episodes > 1). It is unlikely this produced a false-positive benefit of the *constant* cue in freeze rate, as it had the lowest median percent time frozen of any discrete condition. A hybrid cue design, constantly on but changing (in color, size, etc.) with FoG, could feasibly reduce FoG incidence *and* duration. The immediate and long-term efficacy of such a strategy should be investigated and could be compared to constant-only/intermittent-only cues.

Contrary to prior studies, the physical cue did not reduce FoG compared to no cue [9,13,14,23]. This physical cue was designed for and has largely been tested in straight, unobstructed walking tests [9,11,23]. In this course, which included turns and obstacles, only 5.6 % of the 1,285 FoG episodes began in the straight, unobstructed portion. The AR cues, which were designed for multidirectional movement, likely held advantage over the physical cue as turns trigger FoG more frequently than linear gait [4]. Another possible explanation is that exclusion of the physical cue from randomization rendered it more susceptible to an unfamiliarity effect. Overall, it is encouraging that AR cues outperformed the conventional physical cue in this elderly group.

Considering effect size, differences in non-FoG gait metrics were not significant. Previous studies reported mostly positive effects of AR cueing on speed and stride length [9,12,[15], [16], [17],^31^]. Cross-study comparisons are difficult, as there is strong heterogeneity in experimental tasks, cue designs, and headset features.

Several features of this study likely contributed to effects on FoG not seen in prior AR studies [11]. The large vertical field-of-view of the ML2 likely made cue visualization easier compared to older hardware. This is the largest cohort to date in an AR cueing study; difficulties in triggering FoG may require large samples for adequate power [3,32]. Additionally, comparison of multiple AR cueing modalities within the same trial is rare, and in only one other study was user cue preference incorporated [18]. Multidirectional cues are also rare [11].

The importance of individualizing cues may be the most important lesson for future research. Cue responsiveness likely varies with preference, disease state, FoG phenotype, and users’ intrinsic strategies for interacting with cues. In this study, participants were free to use the cues how they saw fit, reflecting a more realistic application of cueing in everyday life. This introduced greater inter-user variability in utilization, influenced by factors such as prior cueing experiences and activation during FoG vs preemptively. Further exploration is needed into how user-intrinsic factors, along with different designs and activation methods, impact efficacy. An array of cueing options should be provided so individuals can use personally optimal methods.

This study has limitations in generalizability. Although all participants experienced OFF medication FoG, and the 34/36 on PD meds at baseline endorsed worse FoG in the OFF state, no distinction was made between ON/OFF state, DBS use, or FoG phenotype due to insufficient power [2]. Additionally, the cohort lacked diversity, consisting mostly of white non-Hispanics. The survey results should be interpreted with caution as they may be influenced by agreeability bias. The question regarding future use of these AR cueing strategies was meant to isolate interest to the cue itself rather than the ML2 device, but included significant stipulations. Also, users may feel differently about daily cueing if they are responsible for setting up/wearing the device, unlike in this experiment where a provider handled setup.

The design also produced limitations. Efficacy of observer-controlled cues depends on observers’ expertise and consistency. Participants were introduced to the intervention shortly before testing, which may not allow sufficient time to eliminate effects of unfamiliarity [11,18]. This could, however, suggest more training with AR cues could yield greater benefits. The opposite may be true; habituation cannot be assessed with this single-visit design. This study was open-label; blinding is challenging in cueing as it is often apparent to user and observer which condition is being tested. Future studies aiming should evaluate effects over time and blind to the greatest possible extent [11]. This trial does *not* establish these cues as viable for real-world use. Though comparisons with small effect sizes were filtered out, multiplicity of testing remains a valid consideration in interpreting findings. Results should be considered within the context of an exploratory trial intended to guide future works.

## 5 Conclusion

In a cohort of 36 with PD and FoG, a novel multidirectional AR cue was tested for efficacy in reducing FoG compared to physical-cue and no-cue control conditions. Four cue modalities were compared: *hand-controlled, eye-controlled, observer-controlled*, and *constant*. The *observer-controlled* cue decreased percent time frozen and the *constant* cue decreased FoG incidence compared to controls. In 28 participants who chose an AR cue as the most effective condition, their preferred AR cue significantly decreased both percent time frozen and freeze rate compared to controls.

## CRediT authorship contribution statement

**Brendan Baugher:** Conceptualization, Data curation, Formal analysis, Funding acquisition, Investigation, Methodology, Supervision, Validation, Visualization, Writing – original draft, Writing – review & editing. **Ryan Kaya:** Investigation, Methodology, Writing – review & editing. **Claire Sonneborn:** Data curation, Formal analysis, Methodology, Supervision, Validation, Writing – review & editing. **Kenneth B. Baker:** Supervision, Validation, Writing – review & editing. **Hubert H. Fernandez:** Conceptualization, Funding acquisition, Methodology, Project administration, Supervision, Validation, Writing – review & editing. **Nathaniel Szewczyk:** Conceptualization, Data curation, Formal analysis, Funding acquisition, Investigation, Methodology, Project administration, Resources, Software, Supervision, Validation, Visualization, Writing – original draft, Writing – review & editing. **Jay L. Alberts:** Investigation, Resources, Software, Writing – review & editing. **James Liao:** Conceptualization, Data curation, Formal analysis, Funding acquisition, Investigation, Methodology, Project administration, Resources, Software, Supervision, Validation, Visualization, Writing – original draft, Writing – review & editing.

## Declaration of competing interest

The authors declare the following financial interests/personal relationships which may be considered as potential competing interests: James Liao reports financial support was provided by American Parkinson Disease Association. Brendan Baugher reports financial support was provided by American Parkinson Disease Association. Brendan Baugher reports financial support was provided by Ohio University Russ College of Engineering and Technology. Brendan Baugher reports financial support was provided by Ohio University Graduate College. Active licensing agreement with Strolll Limited for the techniques described in the manuscript – BB, JL; Editor-in-chief of Parkinsonism and Related Disorders – HHF; Scientific advisory board member for Strolll Limited – JLA If there are other authors, they declare that they have no known competing financial interests or personal relationships that could have appeared to influence the work reported in this paper.
