# Supplementary material for: Effects of augmented reality cueing strategies on freezing of gait: The ELIMINATE FoG trial
Source: Clin Park Relat Disord. 2025 Apr 29;12:100332. doi: 10.1016/j.prdoa.2025.100332 (PMC12099459; doi:10.1016/j.prdoa.2025.100332)
Supplement: Supplementary Data 1 [file mmc1.docx]

**ELIMINATE FoG Sample Size Estimate**

The following sample options were considered in a preliminary estimate:

“Sample size calculations were based on effect sizes reported in Lewis et al 2000 [1], where the difference in stride length (in meters) between a step length marker cue and no cue was reported to be 0.19 (pooled SD = 0.23), and the difference between a subject-mounted light device cue and no cue was found to be 0.24 (pooled SD = 0.17). Sample sizes are based on a two-sided repeated measures ANOVA, where each subject provides data for each of the five intended cueing conditions, and a conservative correlation between repeated measures of 0.2 was applied. To detect the effect size observed between the step length marker cue and no cue, 17 subjects are needed for analysis to provide 70% power, and 21 subjects are needed to provide 80% power. To detect the effect size observed between the subject-mounted light device cue and no cue, 8 subjects are needed for 70% power and 9 subjects are needed for 80% power.

Because of the relatively strict assumptions of repeated measures ANOVA (normality, sphericity, and compound symmetry covariance), and because of anticipated smaller effect sizes among cueing conditions in the proposed study, sample size calculations were replicated with a scaling factor of 0.75 applied to the smaller of the previously-considered mean differences (i.e. mean difference of 0.19 between step length marker and no cue multiplied by 0.75 to yield a mean difference of .14). To detect this difference with 70% power, 29 subjects would be required. 36 subjects would be required to detect the same difference with 80% power."

The largest estimate of n=36 was chosen. All calculations were two-tailed, assumed an alpha level of 0.05, and were carried out using SAS Studio v.3.6. Six conditions were ultimately included in the protocol. ANOVA was used in this estimate solely to provide a conservative preliminary recruiting target, which resulted in the largest cohort in an AR FoG cueing trial to date. Other analyses were ultimately chosen (see *Statistics and Reproducibility* within the manuscript).

The Lewis et al study [1] was chosen as it is a landmark study in establishing the physical cue utilized in the current study, and because no prior AR cueing trials observed significant findings in percent time frozen or freeze rate (suggesting potential underpowering).

[1] G.N. Lewis, W.D. Byblow, S.E. Walt, Stride length regulation in Parkinson’s disease: the use of extrinsic, visual cues, Brain 123 (2000) 2077–2090. <https://doi.org/10.1093/brain/123.10.2077>.
