# Supplementary material for: Effects of augmented reality cueing strategies on freezing of gait: The ELIMINATE FoG trial
Source: Clin Park Relat Disord. 2025 Apr 29;12:100332. doi: 10.1016/j.prdoa.2025.100332 (PMC12099459; doi:10.1016/j.prdoa.2025.100332)
Supplement: Supplementary Data 3 [file mmc3.docx]

| Supplemental Table 1: Primary outcome medians (interquartile range in parentheses~~)~~ [AR=augmented reality] | | | | | | |  |  |  |
| --- | --- | --- | --- | --- | --- | --- | --- | --- | --- |
| Full Cohort | | | | | | | Preferred AR Cue Subgroup | | |
|  | No cue | Physical cue | Constant cue | Hand-controlled cue | Eye-controlled cue | Observer-controlled cue | Preferred AR cue | No cue | Physical cue |
| Percent time frozen | 5.30% (2.51%,37.3%) N=36 | 6.54% (2.67%,17.1%)  N=36 | 2.64% (0.01%,24.66%)  N=36 | 6.09% (1.63%,20.41%)  N=36 | 4.78% (2.09%,22.47%)  N=36 | 3.66% (1.34%,15.30%)  N=36 | 2.27% (0.62%,13.60%)  N=28 | 4.34% (1.93%,22.06%)  N=28 | 7.47% (2.51%,17.14%)  N=28 |
| Freeze rate in episodes/minute | 1.69 (1.16,2.64)  N=36 | 1.87 (0.91,2.85)  N=36 | 1.14 (0.69,2.60)  N=36 | 1.78 (0.69,2.60)  N=36 | 1.71 (0.94,3.04)  N=36 | 1.65 (0.74,3.33)  N=36 | 1.02 (0.47,3.04)  N=28 | 1.67 (1.03,2.64)  N=28 | 1.87 (0.83,2.73)  N=28 |
