# Supplementary material for: Effects of augmented reality cueing strategies on freezing of gait: The ELIMINATE FoG trial
Source: Clin Park Relat Disord. 2025 Apr 29;12:100332. doi: 10.1016/j.prdoa.2025.100332 (PMC12099459; doi:10.1016/j.prdoa.2025.100332)
Supplement: Supplementary Data 4 [file mmc4.docx]

| Supplemental Table 2: Wilcoxon signed~~-~~rank results for primary outcomes (events with p≤.05 and \|Wilcoxon effect size\|>0.10 in bold) [WES=Wilcoxon effect size, MoD=median of differences (interquartile range in parentheses)] | | |
| --- | --- | --- |
|  | Percent time frozen | Freeze rate |
| Comparing the no~~-~~cue control to the: |  |  |
| Constant cue | N=36, p=0.076, z=1.76, WES=0.30, MoD=0.70% (-3.79%,5.18%) | **N=36, p=0.001, z=3.27, WES=0.54, MoD=0.56 (-0.52,1.64)** |
| Hand-controlled cue | N=36, p=0.239, z=1.18, WES=0.20, MoD=0.62% (-4.38%,5.61%) | N=36, p=0.087, z=1.71, WES=0.29, MoD=0.26 (-0.94,1.39) |
| Eye-controlled cue | N=36, p=0.271, z=1.10, WES=0.18, MoD=0.56% (-3.91%,5.03%) | N=36, p=0.637, z=0.47, WES=0.08, MoD=0.17 (-1.25,1.59) |
| Observer-controlled cue | **N=36, p=0.004, z=2.91, WES=0.48, MoD=1.20% (-2.55%,4.94%)** | N=36, p=0.109, z=1.60, WES=0.27, MoD=0.24 (-1.04,1.51) |
| Physical cue | N=36, p=0.405, z=-0.83, WES=0.14, MoD=~~-~~1.33% (-8.20%,5.54%) | N=36, p=0.975, z=0.03, WES=0.01, MoD=0.02 (-1.40,1.44) |
| Preferred cue | **N=28, p=0.004, z=2.87, WES=0.48, MoD=1.53% (-4.18%,7.25%)** | **N=28, p=0.022, z=2.30, WES=0.38, MoD=0.43 (-0.55,1.41)** |
| Comparing the physical cue to the: |  |  |
| Constant cue | **N=36, p=0.027, z=2.21, WES=0.37, MoD=2.03% (-2.53,6.60%)** | **N=36, p=0.003, z=3.00, WES=0.50, MoD=0.75 (-0.43,1.93)** |
| Hand-controlled cue | N=36, p=0.057, z=1.90, WES=0.32, MoD=1.21% (-2.73,5.15%) | N=36, p=0.074, z=1.79, WES=0.30, MoD=0.16 (-1.26,1.59) |
| Eye-controlled cue | **N=36, p=0.011, z=2.54, WES=0.42, MoD=2.63% (-2.67%,7.94%)** | N=36, p=0.793, z=0.26, WES=0.04, MoD=0.00 (-2.07,2.07) |
| Observer-controlled cue | **N=36, p=0.003, z=2.96, WES=0.49, MoD=3.14% (-3.77%,10.05%)** | N=36, p=0.287, z=1.06, WES=0.18, MoD=0.15 (-1.40,1.70) |
| Preferred cue | **N=28, p=0.002, z=3.17, WES=0.53, MoD=2.08% (-4.43%,8.59%)** | **N=28, p=0.009, z=2.62, WES=0.44, MoD=0.41 (-1.15,1.98)** |
| Comparing the constant cue to the: |  |  |
| Hand-controlled cue | N=36, p=0.310, z=-1.02, WES=0.17, MoD=0.53% (-3.97%,2.90%) | **N=36, p=0.012, z=-2.51, WES=0.42, MoD=-0.45 (-1.64,0.74)** |
| Eye-controlled cue | N=36, p=0.974, z=-0.03, WES=0.01, MoD=0.02% (4.25%,4.22%) | **N=36, p=0.002, z=-3.11, WES=0.52, MoD=-0.44 (-1.72,0.83)** |
| Observer-controlled cue | N=36, p=0.385, z=0.87, WES=0.14, MoD=0.08% (-2.75%,2.91%) | **N=36, p=0.016, z=-2.41, WES=0.40, MoD=-0.13 (-1.41,1.15)** |
| Comparing the hand-controlled cue to the: |  |  |
| Observer-controlled cue | **N=36, p=0.006, z=2.78, WES=0.46, MoD=1.69% (-2.01%,5.39%)** | N=36, p=0.388, z=-0.86, WES=0.14, MoD=-0.02 (-0.80,0.77) |
| Comparing the eye-controlled cue to the: |  |  |
| Observer-controlled cue | N=36, p=0.128, z=1.52, WES=0.18, MoD=0.26~~% (~~-3.39%,3.92%) | N=36, p=0.422, z=0.80, WES=0.13, MoD=0.21 (-0.95,1.36) |
